# Gynecological problems and the health of University Students

**Published:** 2019

**Affiliations:** 1 Ayesha Shahid, Student of MBBS final year 2019, Jinnah Sindh Medical University, Karachi, Pakistan. E-mail: shahidayesha56@yahoo.com

This refers to the manuscript by Akarsu RH, and Alsac SY entitled Risks with Gynecological problems and the health of University Students published in Pak J Med Sci. 2019;35(3):758-763.

The objective of this study is not clear to the readers. The text has too much ambiguity with the terminologies of “gynecological problems”,” gynecological disorders”, “risk with gynecological disorders” and thus the readers are not able to differentiate in them. Authors did not question if their participants have ever been sexually active, about their fertility status, the number of children that the participants have/had, the future intention about having children, whether they are suffering from psychiatric or physical illness; which can have adverse effects on the reproductive health of women, and about the pap smears. There are places where authors could replace the term “risk management” to make their context more clear. The authors have also failed to objectify if they are looking for the risky “*behaviours*” associated with adverse effects on the reproductive health of women or they are predicting the impact of reproductive and gynecological “*symptoms*” on their future respective health. Thus the ambiguity in the objective reflects on the conclusion and fails to produce any desired impact.

